# Downstream effectors of light- and phytochrome-dependent regulation of hypocotyl elongation in *Arabidopsis thaliana*

**DOI:** 10.1007/s11103-013-0029-0

**Published:** 2013-03-01

**Authors:** Sookyung Oh, Sankalpi N. Warnasooriya, Beronda L. Montgomery

**Affiliations:** 1 Department of Energy-Plant Research Laboratory, Michigan State University Plant Biology Laboratories, 612 Wilson Road, Rm. 106, East Lansing, MI 48824-1312 USA; 2 Department of Biochemistry and Molecular Biology, Michigan State University, 603 Wilson Road, Room 212, East Lansing, MI 48824-1319 USA; 3 Present Address: Donald Danforth Plant Science Center, 975 North Warson Road, St. Louis, MO 63132 USA

**Keywords:** De-etiolation, Phytochrome, Hypocotyl, Photomorphogenesis, Arabidopsis, Development

## Abstract

Arabidopsis, like most plants, exhibits tissue-specific, light-dependent growth responses. Cotyledon and leaf growth and the accumulation of photosynthetic pigments are promoted by light, whereas hypocotyl growth is inhibited. The identification and characterization of distinct phytochrome-dependent molecular effectors that are associated with these divergent tissue-specific, light-dependent growth responses are limited. To identify phytochrome-dependent factors that impact the photoregulation of hypocotyl length, we conducted comparative gene expression studies using Arabidopsis lines exhibiting distinct patterns of phytochrome chromophore inactivation and associated disparate hypocotyl elongation responses under far-red (FR) light. A large number of genes was misregulated in plants lacking mesophyll-specific phytochromes relative to constitutively-deficient phytochrome lines. We identified and characterized genes whose expression is impacted by light and by phyA and phyB that have roles in the photoregulation of hypocotyl length. We characterized the functions of several identified target genes by phenotyping of T-DNA mutants. Among these genes is a previously uncharacterized *LHE* (*LIGHT*-*INDUCED HYPOCOTYL ELONGATION*) gene, which we show impacts light- and phytochrome-mediated regulation of hypocotyl elongation under red (R) and FR illumination. We describe a new approach for identifying genes involved in light- and phytochrome-dependent, tissue-specific growth regulation and confirmed the roles of three such genes in the phytochrome-dependent photoregulation of hypocotyl length.

**Electronic supplementary material:**

The online version of this article (doi:10.1007/s11103-013-0029-0) contains supplementary material, which is available to authorized users.

## Introduction

Plants exhibit distinct growth and developmental phenotypes in response to light exposure. A number of photoreceptor families have been identified as regulators of light-dependent growth and development including ultraviolet B (UV-B), blue/ultraviolet A (B/UV-A) and red/far-red (R/FR) receptors. The R/FR-absorbing phytochromes are the most extensively studied of the higher plant photoreceptors. Phytochromes are composed of an apoprotein and a covalently attached linear tetrapyrrole chromophore (bilin). Five distinct apoprotein-encoding genes, i.e., *PHYA*–*PHYE*, exist in Arabidopsis (Quail 1994; Sharrock and Quail 1989), whereas a single chromophore species, i.e., phytochromobilin, has been identified (Terry et al. 1993). Phytochromes are linked to the regulation of numerous aspects of light-regulated growth and development, including seed germination, inhibition of hypocotyl elongation, promotion of cotyledon and leaf expansion, accumulation of photosynthetic pigments, flowering and senescence (Franklin and Quail 2010; Kami et al. 2010). Although significant insights into phytochrome activation and upstream signaling events have been reported in recent years, less is known about the downstream molecular factors that lead to the observable phenotypic differences associated with, and often hallmarks of, photomorphogenesis.

Prior studies established transgenic expression of a gene encoding a phytochrome chromophore-degrading biliverdin reductase enzyme, i.e., *BVR*, as an effective method for inducing phytochrome deficiencies in planta (Lagarias et al. 1997; Montgomery et al. 1999, 2001). Furthermore, the use of distinct promoters or enhancer-trap-based tools for localized *BVR* expression is useful for the generation of lines with distinct patterns of tissue-specific phytochrome deficiencies and associated discrete phytochrome-deficient phenotypes (Costigan et al. 2011; Hopkins and Kiss 2012; Montgomery 2009; Warnasooriya and Montgomery 2009; Warnasooriya et al. 2011). Notably, comparative analyses of CAB3::pBVR and 35S::pBVR3 lines indicated that CAB3::pBVR seedlings, which have mesophyll-specific phytochrome depletion, exhibit elongated hypocotyls relative to No-0 WT and constitutive 35S::pBVR3 seedlings under increasing FR fluences (Warnasooriya and Montgomery 2009). The FR-dependent phenotypic differences in hypocotyl lengths between the 35S:pBVR3 and CAB3::pBVR lines, in part, may be due to differences in patterns of BVR accumulation in cotyledons of these two transgenic lines–35S-driven expression has been shown to be vascular enriched in transgenic plant systems (Hraška et al. 2008; Sunilkumar et al. 2002), whereas CAB3-driven expression is mesophyll specific (Mitra et al. 1989). However, we previously proposed that the observed phenotypic differences in these lines is also attributable to the retention of hypocotyl-localized phytochrome signaling in the absence of mesophyll-localized phytochromes in CAB3::pBVR seedlings, which results in the promotion of hypocotyl elongation in the absence of a dominant inhibitory action by mesophyll-localized phytochromes on hypocotyl elongation (Warnasooriya and Montgomery 2009). A prior assertion that a balance between opposing light-dependent growth forces, i.e., an inhibition of hypocotyl elongation and promotion of hypocotyl growth, to obtain optimal seedling height for a given light environment has been made (Parks et al. 2001). In support of this, results from prior studies support cotyledons as the site of phytochrome-dependent photocontrol of hypocotyl growth inhibition (Black and Shuttleworth 1974; Endo et al. 2005; Warnasooriya and Montgomery 2009) and phytochrome-dependent regulation of gene expression in the hypocotyl (Tanaka et al. 2002). Furthermore, a role of hypocotyl- or stem-localized phytochromes in FR detection and promotion of hypocotyl elongation has been recognized (Ballaré et al. 1990).

As our detailed analyses provided evidence that hypocotyl-localized phytochromes are still active in CAB3::pBVR lines (Warnasooriya and Montgomery 2009), we anticipated gaining insight at the molecular level into targeted disruptions in light-dependent signaling that impacts hypocotyl development. To this end, we initiated comparative analyses of a CAB3::pBVR line relative to a constitutive 35S::pBVR line under continuous FR (FRc) to identify differentially accumulating proteins by 1D SDS-PAGE analysis (Oh and Montgomery 2011), as well as transcriptomic analyses that were subjected to knowledge-based gene clustering assessment (Rosa et al. 2010). Based on changes in gene expression found in our microarray data, we chose candidate genes for further characterization in our current study. This investigation has resulted in an expansion of our understanding of molecular effectors that regulate light-dependent hypocotyl elongation in Arabidopsis through the identification of phytochrome-dependent effectors not identified through classic whole plant gene inactivation by insertional mutation or RNAi approaches combined with follow-up transcriptomic and phenotypic analyses. Furthermore, our studies resulted in the identification of a previously uncharacterized *LIGHT*-*INDUCED* *HYPOCOTYL* *ELONGATION 1* (*LHE1*) gene, whose expression is enriched in cotyledons and is phytochrome dependent.

## Materials and methods

### Plant materials

*Arabidopsis thaliana* Col-0 ecotype and T-DNA mutants were obtained from the Arabidopsis Biological Resource Center (ABRC; http://www.biosci.ohio-state.edu/pcmb/Facilities/abrc/abrchome.htm; (Alonso et al. 2003). Transgenic *BVR* lines, i.e., 35S::pBVR3 and CAB3::pBVR2, were previously described (Montgomery et al. 1999; Warnasooriya and Montgomery 2009). T-DNA insertion mutants *phyA* (SALK_014575) and *phyB* (SALK_022035) used in these studies were previously isolated and described (Mayfield et al. 2007; Ruckle et al. 2007).

### Light sources

White, far-red, and red light sources were those previously described (Warnasooriya and Montgomery 2009). We measured Wc and Rc fluence rates using a LI-250A Light Meter (LI-COR) connected to a LI-COR quantum sensor. FRc fluence rates were measured using a StellarNet EPP2000 spectroradiometer (Apogee Instruments).

### RNA extraction

Total RNA was isolated from 7-day-old whole seedlings grown at 22 °C in a temperature- and humidity-controlled growth chamber with FRc illumination (5 μmol m^−2^ s^−1^) or Rc illumination (50 μmol m^−2^ s^−1^) using RNeasy^®^ Plant Minikit (Qiagen, CA) according to the manufacturer’s instructions and including on-column DNase treatment (Qiagen, CA) for RT-PCR analyses. The quantity of RNA for each sample was analyzed by spectrometry (NanoDrop1000, Thermo Scientific, MA).

### Gene expression analysis using Arabidopsis ATH1 arrays

Seeds of No-0 WT, 35S::pBVR3 and CAB3::pBVR2 were sterilized as previously described (Warnasooriya and Montgomery 2009). Seeds were planted in petri dishes on agar media prepared as described (Warnasooriya and Montgomery 2009). To synchronize germination, plates with seeds were exposed to R light of 75 μmol m^−2^ s^−1^ for 5 min and imbibing seeds were cold-stratified at 4 °C in darkness for 3 days. Seedlings were grown under continuous far-red illumination for 7 days. Seven-day-old vegetative whole seedlings (300–500 mg) were quickly (<1 min) harvested and immediately frozen in liquid nitrogen inside the FR chamber. Total RNA was isolated using an RNeasy^®^ Plant Minikit (Qiagen, CA) according to the manufacturer’s instructions. After assessing the quality of isolated RNA on an Agilent 2100 Bioanalyzer (Agilent Technologies, Inc., CA), we used 500 ng of total RNA to synthesize amplified RNA (aRNA) using a MessageAmp™ Premier RNA Amplification Kit (Applied Biosystems/Ambion, TX) according to the manufacturer’s instructions with the following modifications. For in vitro transcription reaction, samples were incubated for 8 h at 40 °C. Binding of aRNA to magnetic beads following addition of 100 % ethanol was carried out for 5 min with gentle shaking. To capture the magnetic beads-aRNA complex, we placed U bottom plates on a magnetic stand for ~6 min. aRNA was eluted off of the magnetic beads through vigorous shaking for ~7 min. The quality of biotin-labeled aRNA was determined on an Agilent 2100 Bioanalyzer. We submitted ~12 μg of labeled aRNA to the Research Technology Support Facility at Michigan State University for hybridization with a GeneChip^®^ Arabidopsis ATH1 Genome Arrays (Affymetrix, Inc., CA) and acquisition of scanned probe arrays. Each labeled aRNA sample was hybridized to an individual ATH1 Genome Array and microarray analyses were conducted with three independent RNA extractions per sample. The expression data were subjected to per chip normalization (shift to the 75th percentile) with baseline transformation to the median of all samples using GeneSpring GX 10.0 (Agilent Technologies Inc., CA). We then filtered the data on flags (present or marginal in at least 1 of the 9 samples). We performed analysis of variance on log-transformed expression values by applying the Benjamini and Hochberg multiple testing correction with a *p* value cut off of 0.05 across the three groups (No-0 WT, 35S::pBVR3, and CAB3::pBVR2) to control the false discovery rate. Expression data of CAB3::pBVR2 was compared with 35S::pBVR3 to identify genes with changes in gene expression that are associated with the hypocotyl phenotype observed in CAB3::pBVR2 in FRc light. An expression filter of fold change greater or equal to 2.0 was applied for CAB3::pBVR2 samples against the 35S::pBVR3 sample, resulting in 712 genes for further analysis. The full microarray data has been submitted to the NCBI Gene Expression Omnibus database (submission number GSE38989) in a Minimum Information about a Microarray Experiment (MIAME) compliant standard.

### RT-PCR

Oligo(dT)_15_ primed-first-strand cDNA was synthesized from 1 μg of total RNA using a Reverse Transcription System (Promega, WI) according to the manufacturer’s instructions, with the following modifications: RT reactions were incubated at 42 °C for 1 h followed by 95 °C for 5 min and 4 °C for 5 min. The product of first-strand cDNA synthesis reactions was used as template in PCR reactions conducted with GoTaqGreen (Promega, WI). Oligonucleotides for selected genes and an ubiquitin-conjugating enzyme 21 (*UBC21*) as a control gene were designed using AtRTPrimer (Han and Kim 2006). PCR amplification was carried out with gene-specific oligonucleotides (Table 1) at 10 μM. The following thermal cycling conditions were used for the PCR amplification: (1) 1 cycle of denaturation at 94 °C for 2 min; (2) 27 cycles of denaturation at 94 °C for 30 s, annealing at 56 °C for 45 s, extension at 72 °C for 10–40 s; and (3) final extension at 72 °C for 5 min. PCR products were visualized after agarose gel electrophoresis and staining with ethidium bromide.

**Table 1:** Primer sequences for transcript analyses

AGI number	Annotation	Primers (forward/reverse)	Purpose
*At2g36145*	*LHE1*	tgctccaagttcttccgactaatgg/tcccggaggtgctttatctttcttc	RT-PCR
*At2g36145*	*LHE1*	gcagcgacgctcttcgttcca/cttcacttgccggggactcgc	qRT-PCR
*At5g45820*	*CIPK20*	aggcggtgagctttttgata/acccgagagatcgaatcctt	RT-PCR
*At5g45820*	*CIPK20*	ccaccgcgatctcaaaccgga/ccctcaacgcgctaaggccaa	qRT-PCR
*At1g26220*	*GCN5L*	aggcacaatctccactcctccagc/cgggtcagagacgaacccaagcg	RT-PCR
*At1g26220*	*GCN5L*	gcccgcttgggttcgt/ttgcgtataaaaaccattcctttg	qRT-PCR
*At5g25760*	*UBC21*	ccttacgaaggcggtgtttttcag/cggcgaggcgtgtatacatttg	RT-PCR
*At5g25760*	*UBC21*	caaatggaccgctcttatcaaag/ctgaaaaacaccgccttcgt	qRT-PCR

### Quantitative RT-PCR

For quantitative RT-PCR, cDNA was synthesized as described above with the following modifications: first-strand cDNA synthesis was performed with 100 ng of total RNA using random primers. The cDNA was combined with Fast SYBR^®^ Green Master Mix (Applied Biosystems) and used for quantitative PCR on an ABI 7500 Fast Real-Time PCR System (Applied Biosystems). PCR reactions were performed in three technical and three biological replicates. *UBC21* was used as a control gene for normalization in the experiments. The primers used for quantitative RT-PCR are listed in Table 1.

### Genotyping of T-DNA insertion mutants

We obtained T-DNA insertion mutants from the Salk T-DNA insertion mutant collection (Alonso et al. 2003) and genotyped the mutants by PCR as follows: We harvested leaf tissue from 3-week-old mutant plants grown on soil and extracted genomic DNA using (1) a MasterPure™ Plant Leaf DNA Purification Kit (Epicentre^®^ Biotechnologies) according to the manufacturer’s instructions or (2) a simplified CTAB (Cetyltrimethyl Ammonium Bromide)-extraction procedure (Lukowitz et al. 2000). We assessed homozygosity for T-DNA insertion in each SALK line by PCR using a T-DNA left border primer coupled with a target gene-specific oligonucleotide that anneals to a site downstream of the site of T-DNA insertion, and a pair of target gene-specific oligonucleotides that anneal downstream and upstream of the site of T-DNA insertion. PCR amplification was carried out with oligonucleotides at 10 μM. The following thermal cycling conditions were used for the PCR amplification: (1) 1 cycle of denaturation at 95 °C for 2 min; (2) 35 or 38 cycles of denaturation at 95 °C for 1 min, annealing at 55–59 °C for 30 s or 1 min, extension at 72 °C for 1 or 2 min; and (3) final extension at 72 °C for 5 min with a hold at 4 °C. All target gene-specific primers used for genotyping were designed using the web-based SALK T-DNA Primer Design tool (http://signal.salk.edu/tdnaprimers.2.html). Verified homozygous mutant plants were used in subsequent experiments.

### Hypocotyl inhibition assay

To determine whether the T-DNA insertion mutants for candidate genes selected based on microarray analysis displayed impaired hypocotyl development in FRc or Rc, we sterilized and planted seeds of homozygous mutants as described in Warnasooriya and Montgomery (2009). Imbibing seeds were cold-stratified at 4 °C for 3 or 4 days in darkness. Plates were transferred to a humidity-controlled chamber with FRc illumination at indicated fluence rates in μmol m^−2^ s^−1^, Rc illumination at indicated fluence rates or in darkness for 7 days at 22 °C. Using ImageJ software (NIH), we scanned seedlings and used plant images to quantify hypocotyl lengths. The hypocotyl inhibition assay was repeated three times. Percentage dark length and standard deviations of percentage dark length were calculated. We performed unpaired, two-tailed Student’s t-tests to compare the percentage dark length of hypocotyls of mutants relative to cognate wild-type seedlings at each fluence rate.

## Results

### Identification of target genes for mutant analyses

**Fig. 1:**
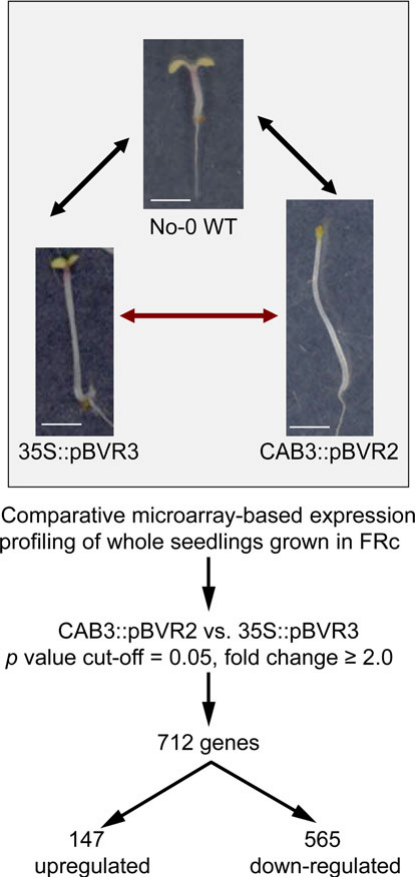
Diagram of the experimental approach used to identify novel genes involved in the far-red dependent regulation of hypocotyl elongation. Images of No-0 WT, 35S::pBVR3 and CAB3::pBVR2 seedlings as described in Warnasooriya and Montgomery (2009) are shown here all at equal magnification to emphasize the relative difference in hypocotyl lengths observed for seedlings grown at 22 °C on Phytoblend medium containing 1 % Suc for 7 days under continuous far-red (FRc) illumination of 5 μmol m^−2^ s^−1^

Under FRc growth conditions, the hypocotyls of the CAB3::pBVR2 line, which exhibits mesophyll-specific inactivation of phytochromes, are significantly longer than hypocotyls of a 35S::pBVR3 line exhibiting constitutive inactivation of phytochromes (Fig. 1; Warnasooriya and Montgomery 2009). Using FRc-grown seedlings, we identified 712 differentially-expressed genes (≥2-fold changes) by GeneSpring^®^ analyses of data from a microarray-based comparison of CAB3::pBVR2 and 35S::pBVR3 lines grown in FRc light (Fig. 1; full MIAME-compliant array data set submitted to Gene Expression Omnibus with submission number GSE38989). The expression of 147 of these genes was upregulated, whereas 565 exhibited downregulation.

**Fig. 2:**
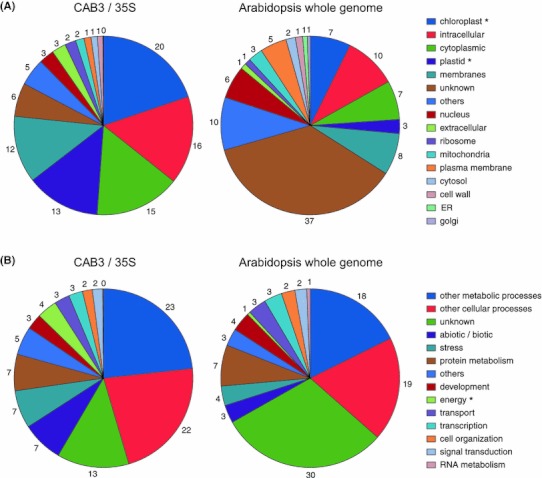
Functional categorization of differentially expressed genes in CAB3::pBVR2 relative to 35S::pBVR3. In a comparison of CAB3::pBVR2 (CAB3) and 35S::pBVR3 (35S) lines, 712 differentially-expressed genes (≥2-fold changes) and ~26,000 genes from Arabidopsis whole genome were grouped by cellular component (**a**) or biological process (**b**) based on Arabidopsis Gene Ontology annotation. Percentages are listed for each category. * indicates a category of interest

The full set of 712 genes was subjected to functional categorization according to gene ontology (GO) annotations using TAIR (www.arabidopsis.org/tools/bulk/go/index.jsp). A larger number of chloroplast/plastid and energy-related genes were differentially expressed in the CAB3::pBVR2 line relative to 35S::pBVR3, as compared to those in the whole genome of Arabidopsis (chloroplast/plastid genes: 33 % in CAB3::pBVR2 vs. 35S::pBVR3, 10 % in Arabidopsis whole genome; energy-related genes: 4 % in CAB3::pBVR2 vs. 35S::pBVR3, 1 % in Arabidopsis whole genome) (Fig. 2). These results suggest that regulation of the expression of chloroplast/plastid and energy-related genes by mesophyll-specific phytochromes is correlated with de-etiolation of Arabidopsis seedlings in FRc light. A number of energy-related genes that were identified among downregulated genes include several genes that encode proteins involved in photosystem structure and function (Table 2), which is correlated with prior studies that demonstrate a reduction in photosynthesis-related *CAB* mRNA in a *phyA* mutant after FR exposure (Reed et al. 1994). We also noted downregulation of genes such as *At5g05270*, which encodes a chalcone flavone isomerase family member (i.e., downregulated ~3-fold), that is correlated with a prior reported phenotype of reduced FRc-associated anthocyanin accumulation in the CAB3::pBVR2 line relative to 35S::pBVR3 (Warnasooriya and Montgomery 2009).

**Table 2:** Energy-related genes misregulated in CAB3::pBVR2 versus 35S::pBVR3 seedlings

Rank^a^	AGI no.	Fold change^b^	Annotation	Reference(s)
4	*At1G70820*	−36.4	Putative phosphoglucomutase	NA^c^
6	*At4G26530*	−25.3	Putative fructose-bisphosphate aldolase	NA
23	*At1G20020*	−7.5	Ferredoxin-NADP(+) oxidoreductase	Lintala et al. (2007)
26	*At2G39470*	−7.1	Photosystem II reaction center PsbP family protein; *PsbP*-*LIKE PROTEIN 2* (*PPL2*)	Ishihara et al. (2007)
29	*At1G70760*	−6.6	*CRR23* (*CHLORORESPIRATORY REDUCTION 23*)	Shimizu et al. (2008)
31	*At2G21330*	−6.4	*FRUCTOSE*-*BISPHOSPHATE ALDOLASE 1* (*FBA1*)	NA
40	*At3G01500.2*	−5.6	*CARBONIC ANHYDRASE 1* (*CA1*)	NA
48	*At1G14150*	−5.0	*PHOTOSYNTHETIC NDH SUBCOMPLEX L 2* (*PNSL2*); *PSBQ*-*LIKE 1* (*PQL1*)	Yabuta et al. (2010)

^a^Rank in list of top 50 of the 565 downregulated genes from most to least downregulated

^b^Level of downregulation of gene expression

^c^NA, not applicable or no published report on function of the indicated gene

In this study, we were interested in identifying genes that are associated with the misregulation of light-dependent hypocotyl elongation. We initially screened T-DNA mutants for ~25 genes whose expression was altered by at least twofold in a comparison of CAB3::pBVR2 and 35S::pBVR3 lines (Supplemental Table S1). In addition to fold change in gene expression, these differentially-expressed genes were identified based on further assessment of information available from functional categorization of GO annotations on TAIR and/or data from publicly available gene expression data sets indicating tissue-specific or light-dependent gene expression patterns (AtGenExpress, www.weigelworld.org/resources/microarray/AtGenExpress). Thus, the ~25 genes were identified as candidates as they represented targets annotated as energy-related, FR-upregulated, tissue-specific, direct targets of LONG HYPOCOTYL5 (HY5), a TF that promotes photomorphogenesis (Osterlund et al. 2000) and that was previously documented as a component of phytochrome-mediated light signaling targets, or putative regulatory factors such as transcription or signaling factors (Supplemental Table S1). Among the lines for which homozygous mutants were isolated, three showed reproducible elongation of hypocotyl lengths under FRc illumination (see bolded lines in Supplemental Table S1). Here, we further characterize the functions of these three down-regulated genes by analyses of available homozygous T-DNA insertion alleles from the Salk Institute Genomic Analysis Laboratory (SALK, http://signal.salk.edu; Table 3).

**Table 3:** Genes for characterization as effectors involved in the photoregulation of hypocotyl elongation

AGI no.	Fold change^a^			Annotation	Expression/function	Mutant lines^b^	Reference(s)
	35S/WT	CAB3/WT	CAB3/35S				
*At2g36145*	−2.3	−11.2	−5.0	*LHE1*	FR^c^	SALK_042596; SALK_051078	Zybailov et al. (2008)
*At5g45820*	−2.1	−15.1	−7.4	*CIPK20*/*SnRK3.6*	HY5-Target/Kinase	SALK_040637; SALK_003410	Gong et al. (2002), Lee et al. (2004)
*At1g26220*	−2.0	−4.0	−2.0	*GCN5L*	N-acetyl transferase	SALK_062388; SALK_150736	Zybailov et al. (2008)

^a^−downregulation of gene expression

^b^Mutant lines. SALK T-DNA insertional mutant lines

^c^FR, upregulated by far-red light treatment

**Fig. 3:**
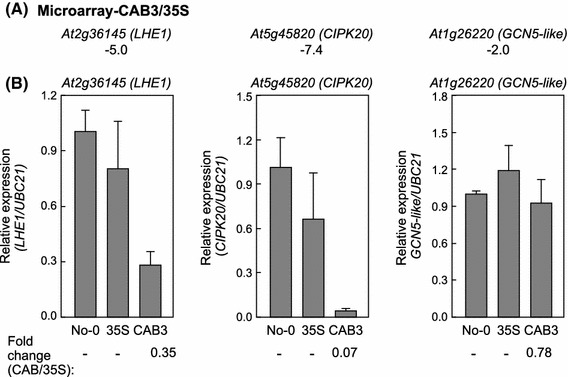
Validation of microarray data. Differentially expressed genes identified by microarray analysis were validated by qRT-PCR using No-0 wild-type, 35S::pBVR3 (35S), and CAB3::pBVR2 lines (CAB3). **a** CAB3 versus 35S, signal intensity for CAB3::pBVR2 over that of 35S::pBVR3 from microarray analysis (fold change). *Negative values* indicate downregulation of gene expression. **b** For qRT-PCR, average relative expression (±SD, *n* = 3) compared to *UBC21* that was used as an internal control is shown. *Numbers* below *graph* in **b** indicate fold-change in gene expression for CAB3 versus 35S

The three candidate genes targeted for additional analysis are as follows: (1) a five-fold down-regulated gene *At2g36145* encoding a chloroplast luminal protein of unknown function (Zybailov et al. 2008) and denoted *LIGHT*-*INDUCED* *HYPOCOTYL* *ELONGATION 1* (*LHE1*), which has been shown to be upregulated in wild-type Arabidopsis 20-fold by FR light treatment at 10 μmol m^−2^ s^−1^ for 4 h compared to dark controls in publically available expression data (AtGenExpress); (2) *At5g45820* encoding a CBL-Interacting Protein Kinase 20 (CIPK20), which is annotated as a kinase and also known as *SnRK3.6* (Gong et al. 2002; Lee et al. 2007), whose expression was downregulated by 7.4-fold based on microarray analysis (Table 3); and (3) *At1g26220* encoding General Control Nonderepressible 5 (GCN5)-Like and thus denoted *GCN5L*, which was twofold downregulated based on comparative microarray-based gene expression profiling (Table 3). Notably, *CIPK20* was also identified as a light regulatory-associated gene in a prior knowledge-based gene clustering analysis of microarray data comparing FRc-grown CAB3::pBVR2 versus 35S::pBVR3 (Rosa et al. 2010). Furthermore, this gene is a HY5 target, the latter of which is a photomorphogenesis-related TF as mentioned above and which we identified as upregulated in our target gene list (Supplemental Data Set 1). CIPK20 was previously shown to upregulated in a *hy5* mutant (Kleine et al. 2007), which corresponds to its being downregulated in the CAB3::pBVR line where *HY5* is upregulated. Using quantitative RT-PCR (qPCR) analyses, we assessed the expression changes that had been determined by microarray analysis for *At2g36145* (*LHE1*), *At5g45820* (*CIPK20*), and *At1g26220* (*GCN5L*) (Fig. 3). These results confirmed the microarray-based expression changes for *LHE1* and *CIPK20* in CAB3::pBVR2 versus 35S::pBVR3. The change observed for *GCN5L* by qRT-PCR was less robust than detected by microarray (0.78 vs. 0.50, respectively; Fig. 3), as were changes in the gene expression for all three genes in a comparison of WT versus 35S::pBVR3.

### Tissue specificity and light regulation of molecular effectors identified in FR-dependent hypocotyl elongation screen

**Fig. 4:**
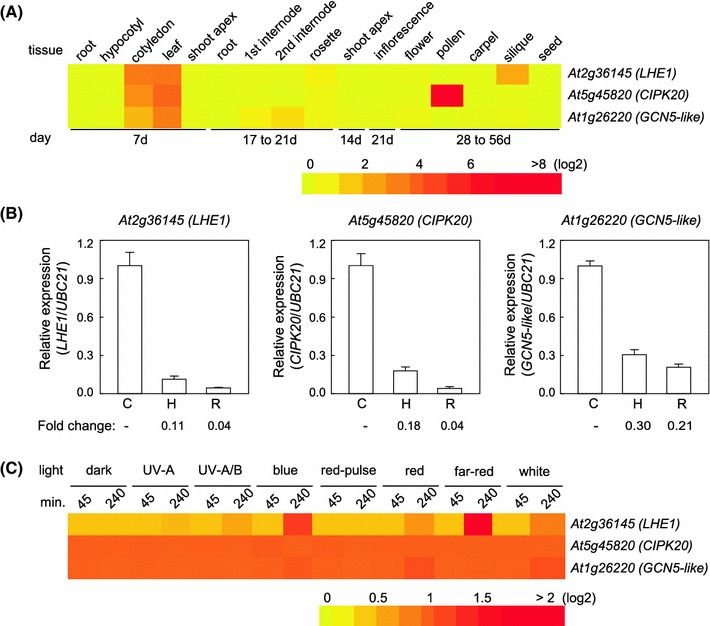
Heat map and qRT-PCR showing the level of candidate gene expression in different Arabidopsis tissues (**a**, **b**) or different light conditions (**c**). Mean-normalized values for Col-0 WT from AtGenExpress expression library (www.weigelworld.org) and BAR Heatmapper Plus (bar.utoronto.ca) were used to construct the heat maps (**a**, **c**). For light experiments, 4-day-old seedlings grown on MS medium under various light conditions for either 45 or 240 min and aerial parts (hypocotyls and cotyledons) were tested. For qRT-PCR (**b**), different tissues from No-0 WT seedlings grown in FRc (4 μmol m^−2^s^−1^) for 7 days were collected and used to extract RNA. *C* cotyledons, *H* hypocotyls, *R* roots. Average relative expression (±SD, *n* = 3) compared to *UBC21* that was used as an internal control is shown. *Numbers* below *graph* in **b** indicate fold-change in gene expression relative to the level of expression in the cotyledon

The analysis of public microarray data from AtGenExpress showed that *At2g36145* or the *LHE1* gene, annotated as an unknown gene, is highly expressed in cotyledon, leaf, and silique tissues (Fig. 4a). Our qRT-PCR analysis showed that *LHE1* mRNA was highly abundant in cotyledons, relative to hypocotyls and roots (Fig. 4b). The *LHE1* gene also has been reported in publically available expression data to be highly upregulated after exposure to 4 h of R (10 μmol m^−2^s^−1^) or FR (10 μmol m^−2^s^−1^) light (Fig. 4c). According to our analysis, this gene was highly upregulated in FRc (5 μmol m^−2^s^−1^) or white light (W, 100 μmol m^−2^s^−1^) in comparison to constant dark treatment for 7-d-old seedlings (Supplemental Fig. S1a). According to publically available expression data, *At5g45820* that encodes CIPK20 is highly expressed in pollen and upregulated in cotyledon and leaf tissues (Fig. 4a). The gene exhibited a slight increase in expression in response to 45 min exposure of R, B or W light (Fig. 4c). We confirmed the cotyledon-enriched expression of *CIPK20* (Fig. 4b). However, in contrast to the AtGenExpress data, we noted that *CIPK20* was highly upregulated in plants irradiated with FRc or W light compared to dark for 7-day-old seedlings (Supplemental Fig. S1b). AtGenExpress public array data indicated that *GCN5L* gene *At1g26220* is highly expressed in cotyledon and leaf tissues (Fig. 4a), and upregulated in response to 4 h exposure of B, R or W light (Fig. 4c). Our RT-PCR analysis showed that *GCN5L* is upregulated in cotyledons (Fig. 4b) and in 7-day-old seedlings exposed to FRc and Wc light (Supplemental Fig. S1c). Notably, FRc upregulation of *GCN5L* expression was previously detected in a microarray analysis (Ma et al. 2001).

### Regulation of candidate genes by phytochromes

**Fig. 5:**
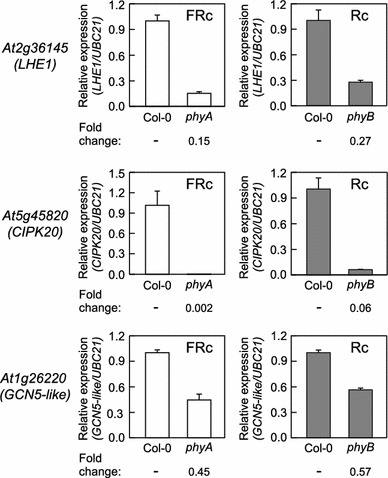
qRT-PCR analysis of candidate genes in *phyA* or *phyB* mutant seedlings. Seven-day-old seedlings of Col-0 WT, *phyA* (SALK_014575), and *phyB* (SALK_022035) were grown at 22 °C on Phytoblend medium containing 1 % Suc under FRc (5 μmol m^−2^ s^−1^) or Rc (50 μmol m^−2^ s^−1^) for RNA extraction to carry out quantitative RT-PCR (qRT-PCR) analysis. Average relative expression (±SD, *n* = 3) compared to *UBC21* that was used as an internal control is shown. *Numbers* below *graphs* indicate fold-change in gene expression for *phy* mutant versus Col-0 WT

To test whether our candidate genes of interest are likely involved in phyA- and/or phyB-mediated light signaling pathways, we examined the expression of these genes in a *phyA* or *phyB* mutant. The expression of genes encoding LHE1, CIPK20, and GCN5L was significantly reduced in both *phyA* and *phyB* mutants in qRT-PCR analyses (Fig. 5). These results indicate a necessary role for phyA and phyB in positive regulation of the expression of these candidate genes.

### Molecular effectors that impact hypocotyl elongation under FRc and continuous red (Rc) illumination

**Fig. 6:**
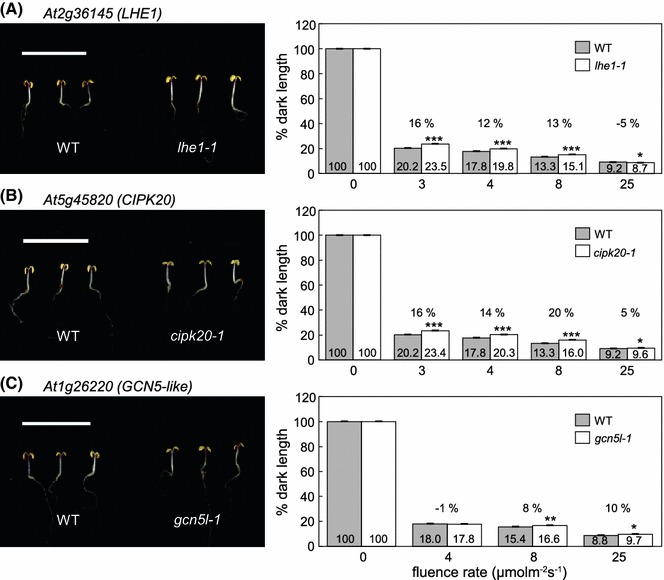
Hypocotyl assay for far-red (FR) light-grown Col-0 WT and SALK T-DNA mutant seedlings. Seedlings with mutations in genes **a** *At2g36145* (*LHE1*), **b** *At5g45820* (*CIPK20*), or **c** *At1g26220* (*GCN5L*) were grown at 22 °C on Phytoblend medium containing 1 % Suc for 7 days under continuous FR illumination at the indicated fluence rate (μmol m^−2^ s^−1^) or in *darkness*. Images to *left* in *each panel* were grown under FRc at 4 μmol m^−2^s^−1^. *Scale bars* indicate 1 cm. *Bars* in *bar graphs* represent mean hypocotyl lengths of seedlings from three independent measurements as a percentage of dark length (±SD). Percentage dark length (*numbers* on *bars*) and percentages of change in hypocotyl elongation relative to WT (*number* above *bars*) are shown. *n* ≥ 75. Unpaired, two-tailed Student’s *t* test comparing mutant to WT for each fluence rate, * *p* < 0.01; ** *p* < 0.005; *** *p* < 0.0001

**Fig. 7:**
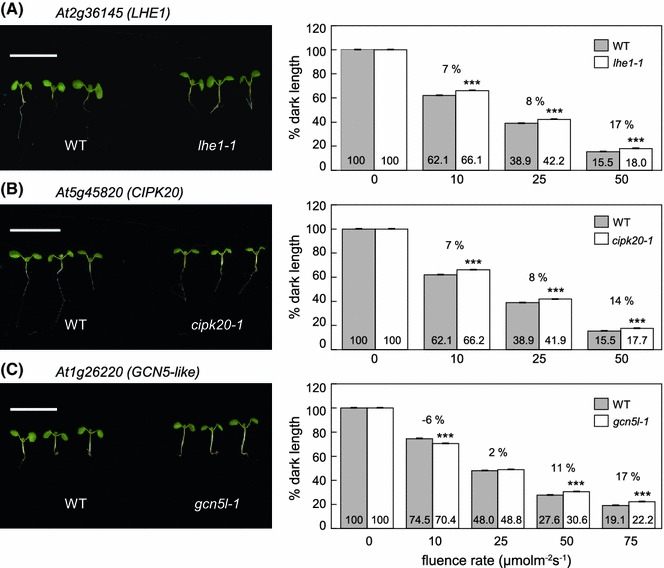
Hypocotyl assay for red (R) light-grown Col-0 WT and SALK T-DNA mutant seedlings. Seedlings with mutations in genes **a** *At2g36145* (*LHE1*), **b** *At5g45820* (*CIPK20*), or **c** *At1g26220* (*GCN5L*) were grown at 22 °C on Phytoblend medium containing 1 % Suc for 7 days under continuous red (Rc) illumination at the indicated fluence rate (μmol m^−2^ s^−1^) or in darkness. Images to *left* in *each panel* were grown under Rc at 50 μmol m^−2^s^−1^. *Scale bars* indicate 1 cm. *Bars* in *bar graphs* represent mean hypocotyl lengths of seedlings from three independent measurements as a percentage of dark length (±SD). Percentage dark length (*numbers* on *bars*) and percentages of change in hypocotyl elongation relative to WT (*number* above *bars*) are shown. *n* ≥ 75. Unpaired, two-tailed Student’s *t* test comparing mutant to WT for each fluence rate, *** *p* < 0.0001

We identified homozygous mutant lines for *LHE1*, *CIPK20*, and *GCN5L* for additional phenotypic analyses. We verified the T-DNA insertion site of each mutant line by PCR using a T-DNA left border primer coupled with a target gene-specific primer that anneals to a site downstream of the site of T-DNA insertion, and a pair of target gene-specific primers downstream and upstream of the insertion (for primer sequences see Supplemental Table S2). We confirmed transcriptional downregulation of the *LHE1* gene in the T-DNA mutant SALK_042596 or *lhe1*-*1* by RT-PCR (Supplemental Fig. S2a). The *lhe1*-*1* mutant displayed significantly elongated hypocotyls, i.e., ~12–16 % longer than Col-0 WT (*p* < 0.0001), under several fluence rates of FRc (Fig. 6a). To determine whether the increase in hypocotyl length observed for the mutant was due to a specific disruption in phyA-mediated hypocotyl inhibition under FRc or whether phytochrome-mediated regulation of hypocotyl inhibition under R light was similarly impacted, the hypocotyl inhibition response was quantified in the mutant relative to Col-0 WT under Rc. The *lhe1*-*1* mutant exhibited ~7–17 % longer hypocotyls than Col-0 WT under Rc of increasing fluence rates (*p* < 0.0001; Fig. 7a). The *lhe1*-*1* mutant was morphologically similar to Col-0 WT, with the exception of the light-dependent increase in hypocotyl length. A second independent *lhe1* allele SALK_051078 (i.e., *lhe1*-*2*), which has reduced expression of the *LHE1* gene (Supplemental Fig. S2a), also exhibited an elongated hypocotyl under FRc light (Supplemental Fig. S3a) and Rc light (Supplemental Fig. S4a).

In SALK_040637, a T-DNA is inserted in the promoter region of *CIPK20* and we confirmed a reduced level of expression of *CIPK20* in this T-DNA mutant (Supplemental Fig. S2b). The *cipk20*-*1* mutant showed significant elongation of hypocotyls in response to FRc light (~14–20 %, *p* < 0.0001, Fig. 6b) and elongation (~7–14 %, *p* < 0.0001) in response to Rc light compared to Col-0 WT (Fig. 7b), indicating that CIPK20 contributes to the inhibition of hypocotyl elongation in FR and R light. Another SALK line with a T-DNA insertion in the promoter region of *At5g45820* (i.e., SALK_003410 or *cipk20*-*2*) also showed a reduced level of *CIPK20* transcript accumulation (Supplemental Fig. S2b) and elongated hypocotyls in response to both FRc light (11–17 %, *p* < 0.0001, Supplemental Fig. S3b) and Rc light (9–16 %, *p* < 0.0001, Supplemental Fig. S4b), supporting a role for CIPK20 in hypocotyl development in response to FR and R light.

SALK_062388 has a T-DNA insertion in the exon close to the start codon of the *GCN5L* gene (Supplemental Fig. S2c). The analysis of *At1g26220* transcript accumulation by RT-PCR in the SALK_062388 or *gcn5l*-*1* mutant indicated that the T-DNA insertion in the exon of *At1g26220* eliminated the accumulation of its transcript (Supplemental Fig. S2c). The analysis of the hypocotyl inhibition response of the *gcn5l*-*1* line showed that the mutant has elongated hypocotyls relative to Col-0 WT hypocotyls under higher fluences of FRc and the increase in the hypocotyl length for *gcn5l*-*1* was ~8–10 % greater than that of Col-0 WT (*p* < 0.01, Fig. 6c). Under Rc, the increase in the hypocotyl length observed for *gcn5l*-*1* was also significant at higher fluences and was ~11–17 % greater than Col-0 WT (*p* < 0.0001, Fig. 7c). A second mutant allele for *At1g26220*, i.e., SALK_150736 or *gcn5l*-*2*, also lacked any transcript accumulation (Supplemental Fig. S2c). This allele likewise exhibited high fluence-dependent elongation under FRc, i.e., ~16 % longer than WT at 25 μmol m^−2^ s^−1^ (*p* < 0.005, Supplemental Fig. S3c). Under Rc, the *gcn5l*-*2* mutant was only significantly longer than WT at 50 μmol m^−2^ s^−1^ (*p* < 0.0001, Supplemental Fig. S4c).

## Discussion

We describe a new approach for identifying novel downstream genes involved in light-dependent, tissue-specific growth regulation. Our work here, as well as prior studies (Costigan et al. 2011; Hopkins and Kiss 2012; Montgomery 2009; Warnasooriya and Montgomery 2009; Oh and Montgomery 2011; Warnasooriya et al. 2011), demonstrated that the use of distinct promoters or enhancer-trap-based tools for localized *BVR* expression to induce phytochrome chromophore degradation results in lines with distinct disruptions in phytochrome-dependent regulation of gene expression and phytochrome-deficient phenotypes. We identified a number of genes whose expression was differentially impacted by localized phytochrome deficiencies using microarray analyses. These transcriptomic-driven analyses of tissue-specific phytochrome-deficient lines resulted in the identification and characterization of phytochrome-dependent effectors not identified through classic mutant isolation, whole plant gene inactivation by insertional mutation or RNAi approaches combined with follow-up transcriptomic and phenotypic analyses. We present data confirming the roles of three such effectors in light- and phytochrome-dependent regulation of hypocotyl elongation. Our data provide evidence that *LHE1* (*At2g36145*) contributes to the inhibition of hypocotyl elongation in response to both FR and R light (Figs. 6, 7). Apposite accumulation of its transcript also requires functional phyA under FRc and phyB under Rc (Fig. 5). It is reported that the protein encoded by *LHE1*, for which there is no prior functional characterization, is enriched in chloroplast thylakoid membranes of Arabidopsis (Peltier et al. 2004). Taken together with our analyses, these findings suggest that regulation of the expression of the gene encoding this chloroplast-localized factor has a role in the light-dependent inhibition of hypocotyl elongation.

Calcium has been implicated as a molecule important for phytochrome signaling (Bowler et al. 1994; Neuhaus et al. 1997). CIPKs, 25 of which have been identified in Arabidopsis, contain a Ser/Thr kinase domain and interact with calcineurin B-like proteins (CBL), a group of calcium-sensing proteins (Kolukisaoglu et al. 2004). Gong et al. (2002) hypothesized that CIPK20 mediates calcium signaling in response to ABA signals and suggested a role for CIPK20 in stress responses. However, it was not determined whether stress-responsive *CIPK20* gene, also designated as *SnRK3.6* based on its classification as a member of the larger CDPK-SnRK superfamily (Hrabak et al. 2003), was involved in phytochrome-mediated inhibition of hypocotyl elongation. Here, we report a disruption in the regulation of hypocotyl elongation in a *cipk20* mutant in FR or R light (Figs. 6, 7). Recently, Qin et al. (2010) demonstrated that salt and ABA-responsive *CIPK14* gene, or *SnRK3.15* (Hrabak et al. 2003), is involved in phyA-mediated FR-dependent block of greening, i.e., a *cipk14* T-DNA mutant grown in FR light did not exhibit any significant greening after irradiation with W light, indicating an enhancement of FR-dependent inhibition of greening in *cipk14* compared to WT (Qin et al. 2010). In addition, the expression level of *CIPK14* was reduced in a *phyA* mutant, suggesting a novel function of CIPK proteins in the phyA signaling pathway (Qin et al. 2010). No impact on FR-mediated inhibition of hypocotyl elongation was reported for the *cipk14* mutant (Qin et al. 2010). Unlike CIPK14, we found that CIPK20 contributes to the inhibition of hypocotyl elongation in both FR and R light (Figs. 6, 7), but not to chlorophyll accumulation (data not shown). These results suggest distinct roles for stress-responsive CIPK proteins in phytochrome-mediated de-etiolation responses, i.e., hypocotyl inhibition versus cotyledon greening. Notably, a line with a mutation in the *SnRK2.5* gene (*At5g63650*; upregulated by twofold in comparative microarray-based gene expression profiling comparing the CAB3::pBVR2 line 35S::pBVR3), another member of the plant-specific SnRK2 subfamily (Halford and Hey 2009), did not have an elongated hypocotyl under FR light (Supplemental Table S1 and data not shown). Thus, *SnRK2.5* likely has a distinct role in Arabidopsis. In this regard, other SnRK2 proteins in Arabidopsis have been implicated recently in ABA-dependent and -independent signaling that impacts responses to osmotic stress (Fujii et al. 2011). Notably, *SnRK2.5* has been recently identified as a direct target of transcriptional regulation by phytochrome-interacting factor (PIF) proteins, i.e., PIF1 and PIF3 (Zhang et al. 2013).

Prior results indicated a short hypocotyl phenotype for a T-DNA insertion mutant of the Arabidopsis GNAT family protein-encoding *GCN5* gene (i.e., *gcn5*-*1*) under Wc light (Vlachonasios et al. 2003), as well as elongated hypocotyls under W, R, FR and B for another *gcn5* mutant allele (Benhamed et al. 2006). Notably, a *gcn5l* mutant, which harbors a mutation in *GCN5L* gene (i.e., *At1g26220*) whose product is purportedly localized to the chloroplast (Zybailov et al. 2008), exhibits elongated hypocotyls under both Rc and FRc light (Figs. 6, 7). The observation of elongated hypocotyls under both light conditions in the *gcn5l* mutants relative to WT suggests that the regulation of the expression of this gene is under both phyA- and phyB-specific signaling control, which contributes to hypocotyl growth inhibition under R and FR.

As a number of molecular effectors are involved in the regulation of hypocotyl development, the contribution of each candidate gene to the overall hypocotyl length can be moderate and thus a small, yet reproducible change in hypocotyl length observed for several individual T-DNA mutant lines suggests that these are valid candidates in the regulation of hypocotyl development in Arabidopsis under Rc and FRc light. In this regard, we have identified a previously uncharacterized gene and novel functions for two known genes. *LHE1*, *CIPK20*, and *GCN5L*, whose expression all appear to be regulated by phyA and phyB, are involved in the light-dependent inhibition of hypocotyl elongation in *A. thaliana*. A change in the expression of any of these genes in the absence of phyA or phyB had only been noted before for *GCN5L*, which was shown to be downregulated more than twofold in a *phyA* null mutant background for seedlings grown under FRc for 6 days (Ma et al. 2001). However, expression changes for the other genes do not show up in microarrays for *phyA* and/or *phyB* mutants (Ma et al. 2001; Tepperman et al. 2004), although the growth conditions under FRc and Rc light ranged from shorter periods, i.e., up to ~24 h (Tepperman et al. 2004) to 6 days (Ma et al. 2001), the latter of which is very similar to growth conditions used in experiments described here. These observations, together with the observation that the target genes are misregulated to different degrees by CAB3::pBVR2 expression compared to 35S::pBVR3 (Supplemental Table S1), indicate that using lines with distinct patterns of phytochrome deficiency facilitate the isolation of unique sets of genes relative to classic mutant lines exhibiting gene deficiency at the level of the whole organism. Furthermore, our results underscore the usefulness of the method described for finding novel components of phytochrome signaling that impact hypocotyl elongation to a lesser degree than might be detected in classic screens for elongated hypocotyl mutants, or that impact phytochrome signaling in a cell- and tissue-specific context. The fact that the expression of these genes is cotyledon-enriched and reduced in phytochrome apoprotein mutants suggests that these factors may be targets of phytochromes in the cotyledon specifically and/or functionally involved in cotyledon-to-hypocotyl signaling.

Notably, each of the characterized genes is likely to have a unique impact in regard to altering tissue-specific, phytochrome-dependent signaling. GCN5 is a histone acetyltransferase that impacts light-dependent gene expression (Benhamed et al. 2006). Thus, GCN5L likely functions similarly to modify histone acetylation and thereby activate gene expression in a tissue-specific, light-dependent fashion that alters the activities of other genes that ultimately regulate hypocotyl elongation. CIPK20 is a member of the CIPK family of genes implicated in calcium signaling (for review see Luan 2009). Thus, CIPK20 is likely a factor involved in the long recognized calcium-dependent repression of genes expression by phytochromes (Neuhaus et al. 1997). Finally, LHE1 represents a novel chloroplast-localized factor that contributes to the inhibition of hypocotyl elongation in a tissue-specific fashion. Together, our analyses provide compelling evidence for the use of lines exhibiting distinct tissue- and organ-specific phytochrome inactivation as important in identifying and elucidating new molecular effectors in light- and phytochrome-dependent regulation of photomorphogenesis.

## Electronic supplementary material

Below is the link to the electronic supplementary material.

Supplementary material 1 (PDF 678 kb)

Supplementary material 2 (XLS 175 kb)
