# Supplementary material for: Downstream effectors of light- and phytochrome-dependent regulation of hypocotyl elongation in Arabidopsis thaliana
Source: Plant Mol Biol. 2013 Mar 1;81(6):627–40. doi: 10.1007/s11103-013-0029-0 (PMC3597320; doi:10.1007/s11103-013-0029-0)
Supplement: Supplementary file 1 — Supplementary material 1 (PDF 678 kb) [file 11103_2013_29_MOESM1_ESM.pdf]

Supplemental Information

Journal – Plant Molecular Biology

Downstream Effectors of Light- and Phytochrome-Dependent Regulation of Hypocotyl Elongation in *Arabidopsis thaliana*

Sookyung Oh[1,4], Sankalpi N. Warnasooriya[1,3,4#], Beronda L. Montgomery[1,2*]

[1]Department of Energy–Plant Research Laboratory, Michigan State University Plant Biology Laboratories, 612 Wilson Road, Rm. 106, East Lansing, MI 48824-1312

[2]Department of Biochemistry and Molecular Biology, Michigan State University, 603 Wilson Road, Room 212 , East Lansing, MI 48824-1319

[3]Current address: Donald Danforth Plant Science Center, 975 North Warson Road, St. Louis, Missouri 63132 USA.

[4]Contributed equally to this work.

[#]Current address: Donald Danforth Plant Science Center, 975 North Warson Road, Saint Louis, MO 63141

[*]Address correspondence to: Beronda L. Montgomery, Department of Energy–Plant Research Laboratory, Michigan State University, Plant Biology Laboratories, 612 Wilson

Road, Rm. 106, East Lansing, MI, 48824-1312, U.S.A.; Tel: 517 353 7802; e-mail: montg133@msu.edu

Supplemental Data Set 1.

712 genes differentially expressed in CAB3::pBVR2 vs. 35S::pBVR3 seedlings has been uploaded as an .xls file

Table S1. Genes screened by mutant analyses for involvement in FR-dependent hypocotyl length

	**Fold change[a]**							
**AGI No.**	**35S /WT**	**CAB3 /WT**	**CAB3 /35S**	**Annotation**	**Expression**	**Mutant lines[b]**	**Geno[c]**	**Reference(s)**
At1g16720	-1.6	-3.9	-2.5	HCF173	Energy[d]	SALK_035984	NA	(Schult *et al.*, 2007)
At1g18710	+1.5	+4.5	+2.9	MYB47	TF[e]/ HY5-Target	SALK_123009	Hm	(Lee *et al.*, 2007)
At1g23160	+6.4	-2.9	-18.6	Auxin-responsive GH3 family	Auxin-responsive	SAIL_755_E09	Hm	
***At1g26220*[f]**	**-2.0**	**-4.0**	**-2.0**	**GCN5-related**	**N-acetyl transferase**	**SALK_062388**	**Hm**	(Zybailov *et al.*, 2008)
At1g34310	-2.0	-8.7	-4.3	ARF12	TF	SAIL_1161_E12	NA	
At1g52410	+2.7	+16.9	+6.3	TSA1	Calcium ion binding	SALK_151393	Hm	(Suzuki *et al.*, 2005)
At1g56660	+2.5	+5.4	+2.2	Unknown	HY5-Target	SAIL_163_D09	Hm	(Lee *et al.*, 2007)
At1g57770	-1.8	-5.6	-3.0	Amine oxidase	FR[g]/ HY5-Target	SALK_088490	Hm	(Lee *et al.*, 2007; Zybailov *et al.*, 2008)
At1g69230	-1.2	-4.3	-3.6	SP1L2	Tissue[h]	SALK_046854<br>SALK_122584	Hm<br>Hm	(Nakajima *et al.*, 2006)
At1g70760	-1.9	-12.6	-6.6	CRR23	Energy	N404957 (NASC)	NA	(Shimizu *et al.*, 2008)
At1g70820	-1.9	-68.2	-36.4	Phosphoglucomutase	Energy	SALK_036170<br>SALK_095953	Hm<br>Hm	(Egli *et al.*, 2010)
At1g72030	-1.5	-4.5	-3.0	GCN5-related	N-acetyl transferase	SALK_010837	NA	
At2g28630	-1.1	-4.9	-4.6	KCS12	HY5-Target	SALK_062883	Hm	(Joubes *et al.*, 2008)
At2g36145	**-2.3**	**-11.2**	**-5.0**	**Unknown**	**FR**	**SALK_042596**	**Hm**	**(Zybailov *et al.*, 2008)**
At2g38300	-1.6	-5.3	-3.4	MYB-like	TF	SALK_112072	Hm	(Tamura *et al.*, 2010)
At2g46680	2.3	6.7	2.9	ATHB7	TF	SAIL_627_B03	Hm	(Johannesson *et al.*, 2001; Olsson *et al.*, 2004; Söderman *et al.*, 1996)

At3g19850	-1.5	-3.4	-2.3	NPH3-related	Light-responsive	SALK_046122	NA	
At3g62090	+1.8	+4.1	+2.3	PIF6 (PIL2)	TF	SALK_147579	Hm	(Penfield *et al.*, 2010)
At4g02290	+1.6	+8.7	+5.5	AtGH9B13	Hydrolase	SALK_101567	Hm	
At4g23290	-1.9	-126.2	-65.1	Protein kinase family	Kinase	SALK_022512	NA	(Heazlewood *et al.*, 2004)
At4g26530	-1.3	-33.4	-25.3	Fructose aldolase	Energy	WiscDsLox335B01	NA	
At5g45820	**-2.1**	**-15.1**	**-7.4**	**CIPK20/SnRK3.6**	**HY5-Target/ Kinase**	**SALK_040637**	**Hm**	**(Gong *et al.*, 2002; Lee *et al.*, 2007)**
At5g50330	+1.6	+6.3	+3.8	ABC1 family	Kinase	SALK_014753	NA	
At5g60890	+1.1	+3.2	+2.8	ATR1 (MYB34)	TF	SALK_006901	Hm	(Bender and Fink, 1998)
At5g63650	+2.6	+5.3	+2.0	SnRK2.5	Kinase	SALK_075624	Hm	(Boudsocq *et al.*, 2004)
At5g66260	+1.1	+4.0	+3.7	Auxin-responsive	Unknown	SALK_095896	Hm	

[a] +, upregulation of gene expression; -, downregulation of gene expression.

[b] Mutant lines. SALK, SAIL, WiscDsLox or NASC T-DNA insertional mutant lines.

[c] Geno, genotype. Hm, homozygous; NA, homozygous not applicable, only heterozygous or WT identified.

[d] Energy, Energy-related genes.

[e] TF, transcription factor.

[f] Genes in bold type indicate genes that were tested further in this study.

[g] FR, upregulated by far-red light treatment.

[h] Tissue, upregulated in specific tissues, e.g., hypocotyl.

Table S2. Primers for verification of homozygous T-DNA lines

**AGI Number**	**T-DNA mutant lines**	**Primer sequences**
	SALK_042596-LP	AAAACTTTTCGCAGAAGAGGC
At2g36145	SALK_042596-RP	AGAAGACGGAGTTTCTCTGGG
(*LHE1*)	SALK_051078-LP	TCCGTAGCCAAATTTGTTGAC
	SALK_051078-RP	AACCGTCCTTGGATGGTAAAC
	SALK_040637-LP	TTATGGACTTGGCAGATTTGG
At5g45820	SALK_040637-RP	TTTTCGCATCAAAACTATGCC
(*CIPK20*)	SALK_003410-LP	TTATGGACTTGGCAGATTTGG
	SALK_003410-RP	TTTTCGCATCAAAACTATGCC
	SALK_062388-LP	GCAAGAAAGAATGCAGCAAAC
At1g26220	SALK_062388-RP	GGATTAGGGTTCTTCGCTCTC
(*GCN5L*)	SALK_150736-LP	CATTCCTGAATTGCAGGAGAG
	SALK_150736-RP	TGTAAATCCTCAATCAACCGC

Supplemental Figure Legends

Figure S1. RT-PCR analyses of expression of genes *At2g36145 (LHE1*), *At5g45820 (CIPK20*), and *At1g26220 (GCN5-like* or *GCN5L*) in different light conditions. Seven-day-old seedlings of Col-0 WT were grown at 22 ºC on Phytablend medium containing 1% Suc for 7 d under constant darkness (Dc), continuous far-red light (FRc; 5 µmol m^{-2} s^{-1}) or continuous white light (Wc; 100 µmol m^{-2} s^{-1}) for RT-PCR analysis. *UBC21* gene (*) expression was detected by RT-PCR shown as internal control.

Figure S2. RT-PCR analyses for independent primary and secondary SALK T-DNA mutant alleles. Schematic representation of the T-DNA insertion site (inverted triangle) and primer pairs (arrowheads) on each gene are indicated. Black boxes denote exons. (a) *At2g36145* (*LHE1*), (b) *At5g45820* (*CIPK20*), and (c) *At1g26220* (*GCN5L*). RT-PCR was performed using Col-0 WT or T-DNA lines (homozygote). * indicates the *UBC21* gene that was used as an internal control. Results shown are representative of at least two independent biological replicates.

Figure S3. Hypocotyl assay under far-red continuous illumination for second, independent SALK T-DNA mutant alleles for (a) *At2g36145 (LHE1*), (b) *At5g45820 (CIPK20*), and (c) *At1g26220 (GCN5L*). Col-0 WT and SALK T-DNA mutant seedlings were grown at 22 ºC on Phytablend medium containing 1% Suc for 7 d under continuous far-red light at the indicated fluence rate (µmol m^{-2} s^{-1}) or in darkness. Data points

represent mean hypocotyl lengths of seedlings from three independent measurements as a percentage of dark length (±S.D.). Percentage dark length (numbers on bars) and percentages of change in hypocotyl elongation relative to WT (number above bars) are shown. n ≥ 75. Unpaired, two-tailed Student's t test, **, p<0.005, ***, p<0.0001.

Figure S4. Hypocotyl assay under red continuous illumination for second independent SALK T-DNA mutant alleles for (a) *At2g36145 (LHE1*), (b) *At5g45820 (CIPK20*), and *At1g26220 (GCN5L*). Col-0 WT and SALK T-DNA mutant seedlings were grown at 22 ºC on Phytablend medium containing 1% Suc for 7 d under continuous red light at the indicated fluence rate (μmol m^{-2} s^{-1}) or in darkness. Data points represent mean hypocotyl lengths of seedlings from three independent measurements as a percentage of dark length (±S.D.). Percentage dark length (numbers on bars) and percentages of change in hypocotyl elongation relative to WT (number above bars) are shown. n ≥ 75. Unpaired, two-tailed Student's t test, *, p<0.01, ***, p<0.0001.

Supplemental Figure S1

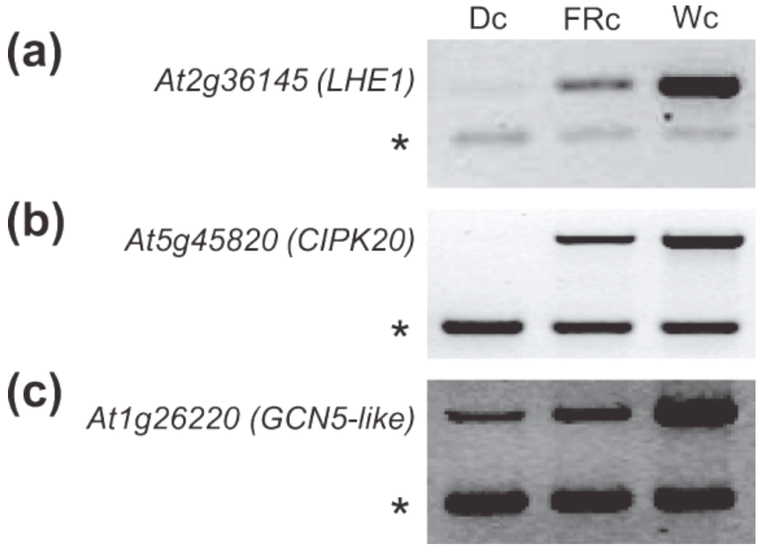

Supplemental Figure S2

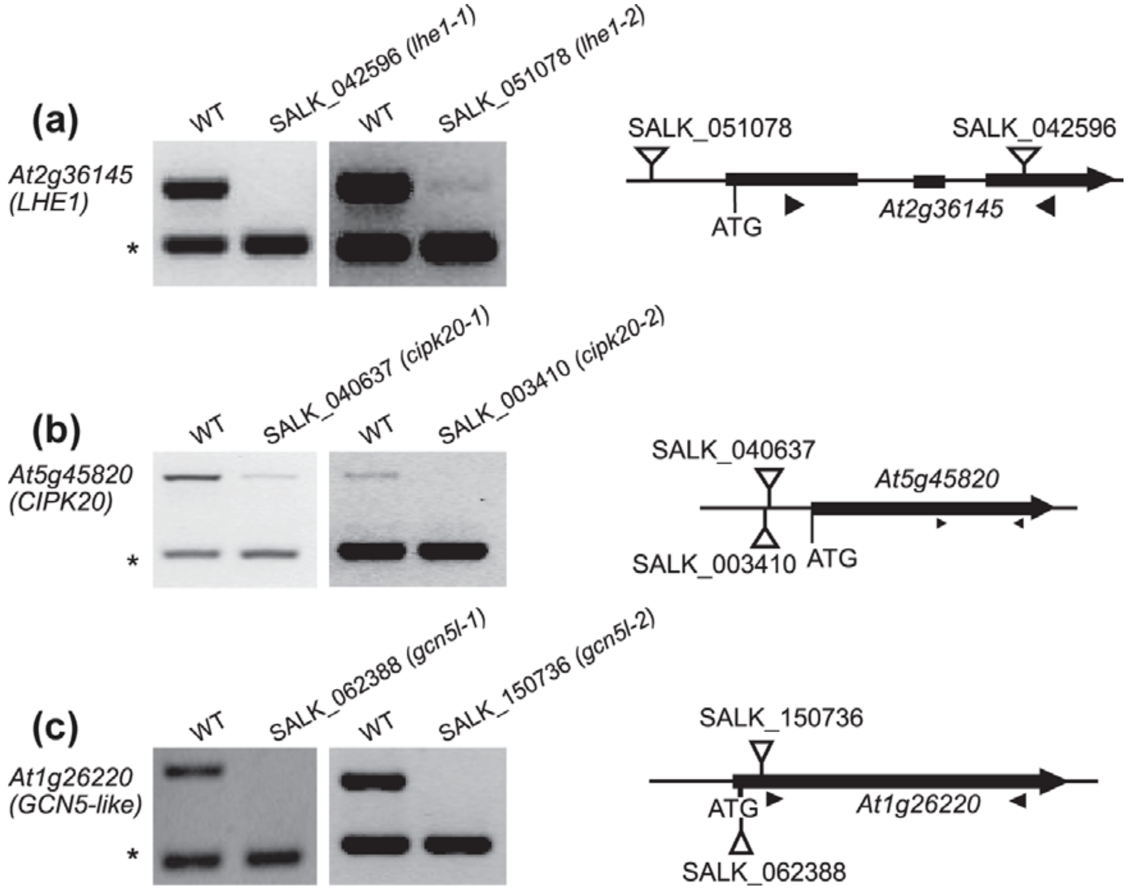

Supplemental Figure S3

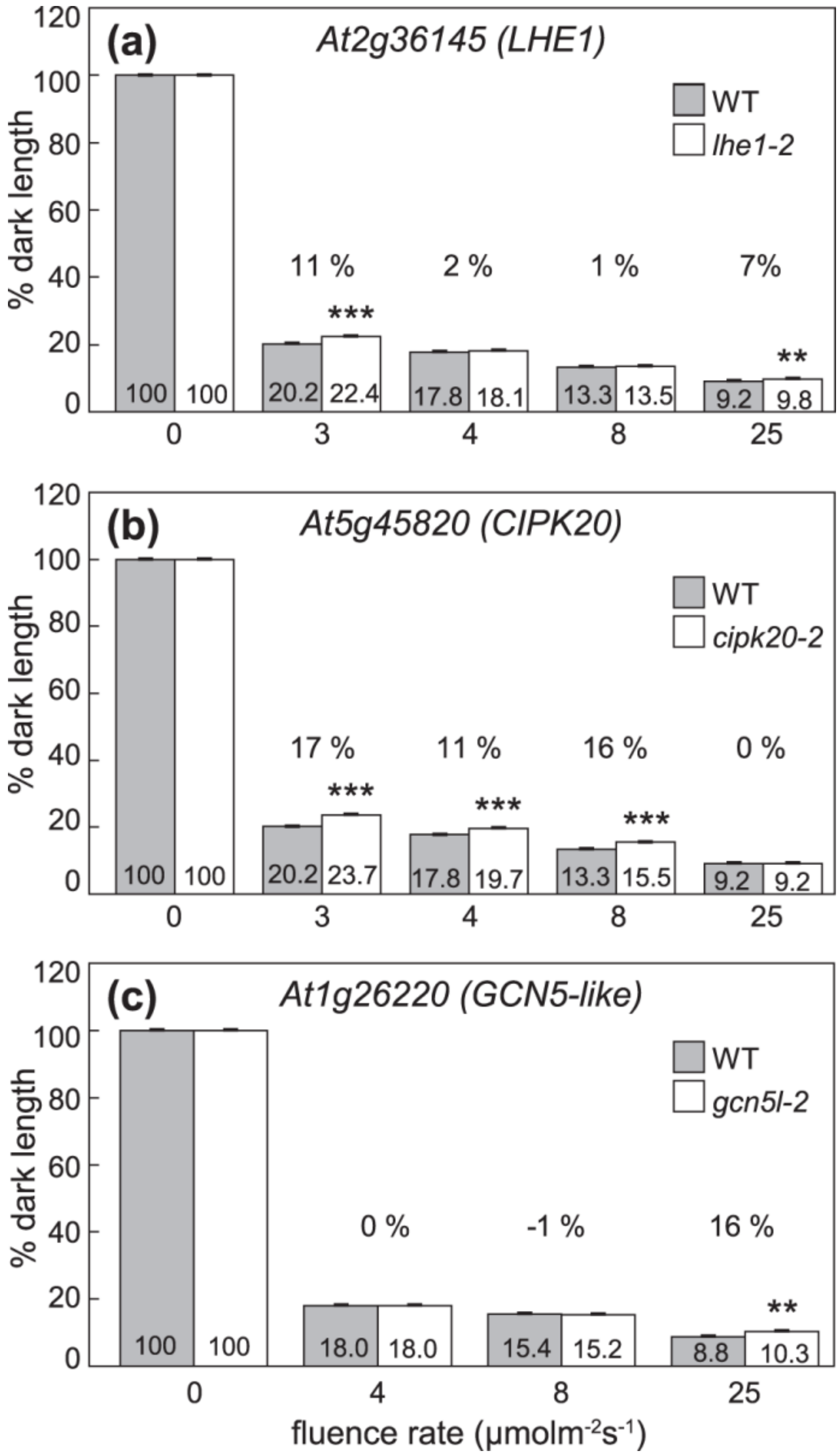

Supplemental Figure S4

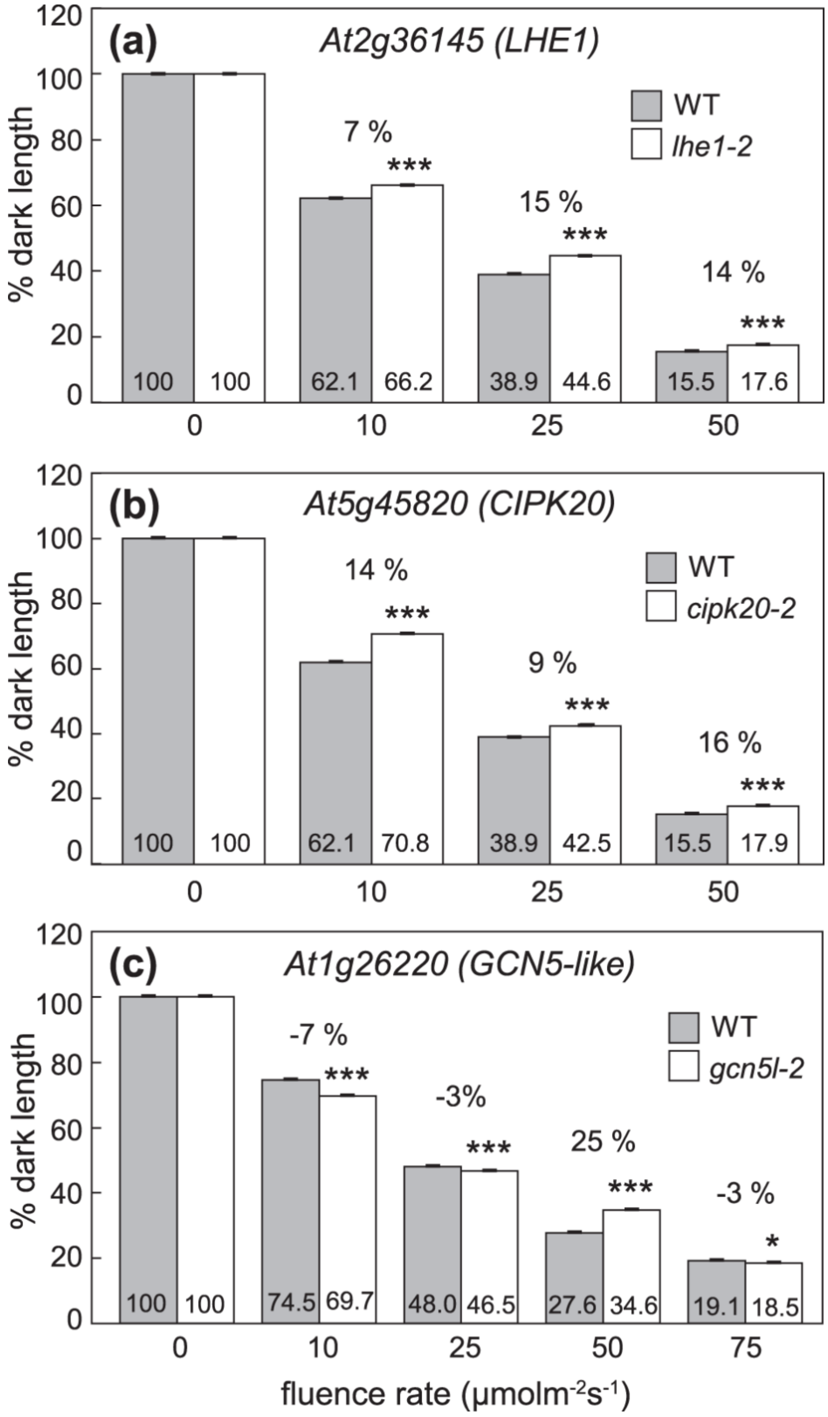

References

Bender J, Fink GR (1998) A Myb homologue, ATR1, activates tryptophan gene expression in Arabidopsis. Proc Natl Acad Sci U S A **95:** 5655-5660

Boudsocq M, Barbier-Brygoo H, Laurière C (2004) Identification of nine sucrose nonfermenting 1-related protein kinases 2 activated by hyperosmotic and saline stresses in *Arabidopsis thaliana*. J Biol Chem **279:** 41758-41766

Egli B, Kölling K, Köhler C, Zeeman SC, Streb S (2010) Loss of cytosolic phosphoglucomutase compromises gametophyte development in Arabidopsis. Plant Physiol **154:** 1659-1671

Gong D, Zhang C, Chen X, Gong Z, Zhu JK (2002) Constitutive activation and transgenic evaluation of the function of an Arabidopsis PKS protein kinase. J Biol Chem **277:** 42088-42096

Heazlewood JL, Tonti-Filippini JS, Gout AM, Day DA, Whelan J, Millar AH (2004) Experimental analysis of the Arabidopsis mitochondrial proteome highlights signaling and regulatory components, provides assessment of targeting prediction programs, and indicates plant-specific mitochondrial proteins. Plant Cell **16:** 241-256

Johannesson H, Wang Y, Engström P (2001) DNA-binding and dimerization preferences of Arabidopsis homeodomain-leucine zipper transcription factors in vitro. Plant Mol Biol **45:** 63-73

Joubes J, Raffaele S, Bourdenx B, Garcia C, Laroche-Traineau J, Moreau P, Domergue F, Lessire R (2008) The VLCFA elongase gene family in Arabidopsis

thaliana: phylogenetic analysis, 3D modelling and expression profiling. Plant Mol Biol **67:** 547-566

Lee J, He K, Stolc V, Lee H, Figueroa P, Gao Y, Tongprasit W, Zhao H, Lee I, Deng XW (2007) Analysis of transcription factor HY5 genomic binding sites revealed its hierarchical role in light regulation of development. Plant Cell **19:** 731-749

Nakajima K, Kawamura T, Hashimoto T (2006) Role of the *SPIRAL1* gene family in anisotropic growth of *Arabidopsis thaliana*. Plant Cell Physiol **47:** 513-522

Olsson AS, Engström P, Söderman E (2004) The homeobox genes *ATHB12* and *ATHB7* encode potential regulators of growth in response to water deficit in Arabidopsis. Plant Mol Biol **55:** 663-677

Penfield S, Josse EM, Halliday KJ (2010) A role for an alternative splice variant of *PIF6* in the control of Arabidopsis primary seed dormancy. Plant Mol Biol **73:** 89-95

Schult K, Meierhoff K, Paradies S, Töller T, Wolff P, Westhoff P (2007) The nuclear-encoded factor HCF173 is involved in the initiation of translation of the *psbA* mRNA in *Arabidopsis thaliana*. Plant Cell **19:** 1329-1346

Shimizu H, Peng L, Myouga F, Motohashi R, Shinozaki K, Shikanai T (2008) CRR23/NdhL is a subunit of the chloroplast NAD(P)H dehydrogenase complex in Arabidopsis. Plant Cell Physiol **49:** 835-842

Söderman E, Mattsson J, Engström P (1996) The Arabidopsis homeobox gene *ATHB-7* is induced by water deficit and by abscisic acid. Plant J **10:** 375-381

Suzuki T, Nakajima S, Morikami A, Nakamura K (2005) An Arabidopsis protein with a novel calcium-binding repeat sequence interacts with TONSOKU/MGOUN3/BRUSHY1 involved in meristem maintenance. Plant Cell Physiol **46:** 1452-1461

Tamura K, Fukao Y, Iwamoto M, Haraguchi T, Hara-Nishimura I (2010) Identification and characterization of nuclear pore complex components in *Arabidopsis thaliana*. Plant Cell **22:** 4084-4097
